# Physiological, Behavioral, and Dietary Characteristics Associated with Hypertension among Kenyan Defence Forces

**DOI:** 10.5402/2013/740143

**Published:** 2013-05-28

**Authors:** Victor Mundan, Margaret Muiva, Samuel Kimani

**Affiliations:** ^1^School of Nursing Sciences, University of Nairobi, P.O. Box 19676 (00202) KNH, Nairobi, Kenya; ^2^Armed Forces Memorial Hospital, Nairobi, Kenya

## Abstract

*Background*. Hypertensive disease is increasing in developing countries due to nutritional transition and westernization. Hypertensive disease among Kenya military may be lower because of health-focused recruitment, physical activities, routine checkups, and health awareness and management, but the disease has been increasing. *Purpose*. The purpose of this study was to determine physiological, behavioral, and dietary characteristics associated with hypertension among Kenyan military. *Methods*. A cross-sectional study involving 340 participants was conducted at Armed Forces Memorial Hospital. Participants' history, risk factors assessment, and dietary patterns were obtained by structured questionnaire, while physiological and anthropometric parameters were measured. *Results*. Hypertensive participants were likely to have higher age, physiological, and anthropometric measurements, and they participated in peace missions. Daily alcohol and smoking, frequent red meat, and inadequate fruits and vegetables were associated with hypertension. *Conclusions*. The findings mimic the main risk factors and characteristics for hypertensive disease in developed countries whose lifestyle adoption is happening fast in low and middle-income countries. Whether or not prediction rules and/or risk scores may identify at-risk individuals for preventive strategy for targeted behavioral interventions among this population require investigation.

## 1. Introduction 

Cardiovascular diseases (CVD), among them hypertensive disease, is causing significant morbidity and mortality in adult population globally [1]. Hypertensive disease is considered in case of untreated systolic blood pressure (BP) of greater than or equal to 140 mmHg and/or diastolic BP of greater than or equal to 90 mmHg or one being on antihypertensive medication [2]. The disease is among the leading causes of death and disability [3, 4], and its prevalence is increasing worldwide [5] particularly in developing countries as a result of nutritional transition [1] and westernization. Hypertensive disease is a significant contributor [6, 7] to the increasing incidence and mortality as a result of CVD globally.

Sub-Saharan Africa appears to bear the heaviest burden of this preventable disease as an emerging threat; for instance, in Cameroon, the prevalence rose from 8.3% in 1994 to 20.8% in 2004 among the adult urban dwellers [8]. In Kenya, available data shows that the disease is gaining momentum and has been identified as an important cause of morbidity and mortality in urban population amidst high prevalence of communicable diseases [9]. The main characteristics and risk factors for hypertensive disease are well established and appear to be consistent across societies. A study among Kenyan Muslim males in Mombasa identified cigarette smoking, alcohol consumption, and overweight as the main risk factors for the disease [10]. Similarly, a study conducted at a leading private hospital in Kenya, hypertensive disease was the main risk factor for ischemic stroke [11]. These studies and others consistently support the magnitude and alarming trend of the disease. The implications are enormous for a health care system that is already struggling with the burden of tropical and communicable diseases including the impact of HIV/AIDs and now the rising noncommunicable diseases. 

Kenyan Defence Forces (KDF) personnel are recruited and retained based on stringent healthy criteria; they undergo routine medical checkups, treatment, and health awareness. Therefore, they may have favorable physical and physiological parameters as indicators of healthy cardiovascular system. Additionally, military personnels maintain strict physical activity schedules and drills regular medical checkups which may result in physical fitness. The military may therefore be healthy with lower rates of hypertension and other CVDs. On the other hand, military personnels practice lifestyle habits similar to the general population which may predispose them to CVD, such as smoking, alcohol intake, and sedentary lifestyle habits. Recently, studies have demonstrated a trend toward increasing hypertensive disease among KDF consistent with other military personnel [12–14] globally. The causation may be multifactorial including occupation-related stress, a potentially important cardiovascular risk factor [15–17]. Military personnels are generally under a lot of pressure and work-related stress, which have been implicated for their biologically negative effects and mental strain [18, 19]. We hypothesized that physiological, behavioral and, dietary characteristics associated with hypertension among KDF mimic the main risk factors and characteristics for CVD in the developed western societies. 

## 2. Methods

This study adopted a cross-sectional design to determine physiological, behavioral, and dietary characteristics associated with hypertension among 340 (170 hypertensive and 170 normotensive) KDF. Eligible participants were examined by trained nurses at the military medical clinic in Armed Forces Memorial Hospital (AFMH), Nairobi. This is a referral hospital that provides healthcare services to the KDF personnel and their nuclear families. It has bed capacity of 114 and 8 specialized outpatient clinics among them a Medical Outpatient Clinic (MOPC). The clinic runs twice a week attending to an average of 40 patients per clinic day. The eligible participants with hypertension were personnels with essential hypertension, on antihypertensive or nonpharmacological therapy for elevated blood pressure based on WHO classification (BP > 140/90 mmHg) as per the hospital file and were being followed up at AFMH MOPC. Similarly, nonhypertensive participants were military personnels recruited from AFMH with normal physiological measurements. The characteristics among the two simple randomly selected sets of participants were compared. Written consent was obtained after comprehensive explanation of study objectives, risks, and benefits. Conventional measurements of physiological as well as anthropometric parameters were conducted by trained nurses thereafter.

*Physiological Variables*. Hypertension was defined according to the Seventh report of the Joint National Committee on Prevention, Detection, Evaluation, and Treatment of high BP criteria as having an untreated systolic BP of greater than or equal to 140 mmHg or diastolic BP greater than or equal to 90 mmHg or being on medication for hypertension [2]. Participants were asked to sit and relax in a silent room for 5 minutes before their arterial pulse and blood pressure were measured in the right arm two times in the sitting or supine position using a calibrated digital instrument, appropriate devices, and cuff sizes. The BP and heart rate were measured with an electronic automatic monitor (OMRON M4, Omron Healthcare Gmbh, Hamburg, Germany). The final pulse and Bp were the average of two consecutive readings obtained from the participant.

*Sociodemographic and Other Behavioural Variables.* Demographic and social life information including age, marital status, level of education, and deployment to peace missions, history of smoking with its frequency were obtained from each individual using a standardized questionnaire given to the participants to complete on their own. 

*Food Intake and Dietary Patterns.* Food consumption data were collected by a self-administered questionnaire. The questionnaire focused on food recall before interview. Participants were asked to report types of foods including meat consumption, beverages such as beer, wine, and whiskey, the frequency of their consumption (daily, weekly, and monthly), and the amounts consumed. 

*Physical Activity.* Participation in sports or exercises was assessed through a questionnaire, and the respondents were asked how often, and the average duration per session they engaged in each of the common physical activities. Individual activities included walking, jogging or running, weight lifting, tennis, soccer, swimming, bicycling, dancing, and other strenuous exercise. The respondents indicated their usual frequency of participation in each of the above activities by choosing one of the following categories: never, less than once per month, 1–3 times per month, 1-2 times per week, 3–6 times per week, or every day. The average time per episode of the activities included less than 15, 16–30, 31–60 min, or more than 60 min, while intensity was categorized as light, moderate, or vigorous.

*Anthropometric Variables.* Anthropometric measures including weight, height, body mass index (BMI), waist circumference (WC), and hip circumference (HC) were measured by standard methods. WC was determined by measuring waist diameter at midpoint between iliac crest and lower border of tenth rib, with an average of three measurements considered as WC. Central obesity was WC > 102 cm and 95 cm as defined for male and female participants, respectively. BMI was calculated based on weight in kilogram divided by square of height in meter (kg/m^2^). Waist and hip circumferences were measured using a nonelastic tape measure (Seca, Germany) and were subsequently used to calculate waist to hip ratio (WHR). Weight and height were obtained using weighing scale with a stadiometer (Seca GmbH, Co.kg, Germany) and, thereafter, used to calculate body mass index (BMI). Height was measured and recorded to the nearest 0.1 cm, while the waist circumference was measured with a nonstretchable tape measure to the nearest 0.1 cm.

## 3. Statistical Analysis

We calculated descriptive statistics (e.g., means, standard deviations, correlations) for each of the major study variables and performed paired *t* tests to determine whether mean scores differed for hypertensive and normotensive participants. Demographic characteristics and potential risk factors were compared by *t*-test for continuous variables and the Chi-square (*χ* ^2^) test for categorical variables. Odds ratios (ORs) were computed by binary logistic regression. Risk estimates were adjusted for age and body mass index (BMI). Association between physiological, dietary patterns, and hypertension by behavioral factors was assessed through subgroup analyses based on different levels of these factors. Adjusting for the effect of all the predictors associated with hypertension was also done. All tests of statistical significance were two-sided, and the analyses were conducted using SPSS for WINDOWS (release 17.0, 2008; SPSS, Chicago, IL, USA). A *P* value of less than 0.05 was considered statistically significant.

## 4. Ethical Consideration

Our research conformed to the principles outlined in the Helsinki declaration. Ethical clearance was obtained from the University of Nairobi/Kenyatta National Hospital Ethics and Research Committee (approval no. P74/03/2011) as well as the Ministry of Education, Science and Technology (permit no. NCST/RRI/12/1/MAS/113). Authorization to carry out the research at AFMH was given by the Armed Forces Memorial Hospital Medical Advisory Committee (approval no. FMH/MED/1).

## 5. Results

### 5.1. Sociodemographic Characteristics

*Gender. *Majority (91.6%) of the study participants were male (Table 1). Most (95%) of participants with hypertension were males compared to 88% of nonhypertensive participants. On further analyses, there were lower odds of being hypertensive among the female gender (OR = 0.44; 95% CI 0.19–1.01, *P* = 0.05).

*Age.* The mean age was 45.1 (SD 7.7) years among participants with hypertensive diseases compared to normotensive 40.8 (7.3) (Table 1). Participants with hypertensive disease were 4.82 years older than nonhypertensive subjects (*P* < 0.01). Majority of hypertensive participants were aged from 39 to 48 years, compared to 14% among normotensive. The odds of being older among participants with hypertension were highest among 49 to 58 years. Additionally, the odds of hypertension in this age group were threefold that of 18 to 28 years (odds ratio [OR] = 3.08; 95% CI 1.2–7.94, *P* = 0.02) age group.

*Deployment to Peace Mission.* We show the relationship between deployment to peace missions and hypertensive disease (Table 2). There was significant (*χ* ^2^ = 34.40; df = 1; *P* < 0.01) association between hypertension and participation in peace missions. Majority (68%) of subjects who had participated in peace missions were hypertensive compared to 36.08% who had never been to peace missions.

*Educational Level.* The association between educational level and hypertension is shown (Table 1). Majority (70%) of participants with hypertensive disease had secondary level education, while most (58.2%) of the normotensive attained tertiary qualification. The majority (87%) of the participants with hypertensive disease appeared to have secondary and primary level qualification.

### 5.2. Lifestyle and Social Behavior

Higher (63%) number of participants with hypertensive disease reported having consumed alcohol compared to 52.07% normotensive subjects (Table 1). Additionally, a strong (*χ* ^2^ = 34.33, df = 3; *P* < 0.01) association between frequency of alcohol consumption and hypertension was established. The most commonly consumed alcoholic drink among hypertensive participants was beer (83.04%) compared to 74.16% among normotensives. Furthermore, the odds of having hypertension among participants who consumed alcohol daily and/or 1 to 3 times per week were significant (OR = 6.29; 95% CI = 0.6–6.77, *P* < 0.01) and (OR = 3.39, 95% CI = 0.2–3.43, *P* < 0.05), respectively. Additionally, the odds of having hypertension among the drinkers of beer or spirits were significant (OR = 1.54; 95% CI = 0.3–1.6, *P* < 0.05) and (OR = 1.2; 95% CI = 0.04–0.13, *P* < 0.05), respectively.

Smoking was prevalent among participants with hypertensive disease compared to normotensives (Table 1). About 11% of hypertensive participants were current smokers compared to 4.2% normotensives. More (32%) participants with hypertension were ex-smokers compared to 18% of normotensives. A strong (*χ* ^2^ = 11.72, df = 2; *P* < 0.01) association was demonstrated between ex-smoking and hypertension.

Additionally, *t*-test analyses showed the smoking duration that is significantly (*P* < 0.05) longer among participants with hypertension compared to normotensives. The average duration of smoking was 2.66 and 2.14 years among hypertensive and nonhypertensive, respectively. The duration of smoking for ex-smokers was significantly (*P* < 0.01) longer for hypertensive compared to the nonhypertensive subjects. The average duration of smoking was 2.6 and 1.8 years among the hypertensive and non hypertensive, respectively.

### 5.3. Dietary Patterns

The relationship between dietary patterns and hypertensive disease was established (Table 1). Our findings showed an association between low intake of fruits and hypertension. There was a significant (*P* < 0.05) association between consuming red meat and hypertension. The frequency of red meat consumption among hypertensive was higher (66.4% daily consumption) compared to nonnormotensive (45.58% daily consumption). 

### 5.4. Level of Physical Activity

The majority (69%) of all participants reported that their work involved lifting weights (Table 2). The participants also reported having participated in cardiorespiratory endurance, flexibility, and muscular endurance as well as muscular strength activities. Our findings showed that 32% of participants with hypertension compared to 21% normotensives were not involved in physical activities. There was a significant (*χ* ^2^ = 9.34, df = 2; *P* = 0.009) association between type of physical activity engaged in and subjects hypertensive status. A higher percentage of normotensives respondents (81.18%) reported participating in muscular endurance exercises compared to 62.94% of hypertensive participants. 

### 5.5. Anthropometric Measures

Participants with hypertensive disease displayed higher anthropometric measurements compared to normotensives (Table 2). They were more likely to be overweight (59.76% versus 28.24%) or obese (19.53% versus 3.53%) compared to normotensives. The association between BMI-defined obesity and hypertension was statistically significant (*P* < 0.01), and only 2.35% of the normotensives had central obesity compared to 32.94% of participants with hypertension (*P* < 0.01).

### 5.6. Physiological Measures

The mean heart rate for participants with no hypertension was lower compared to hypertensive subjects. There was significant (*P* < 0.01) mean difference in heart rate of 6.02 beats/minute between hypertensives (79.91) compared to normotensives (73.89) participants. In addition, participants with hypertension had higher mean systolic as well as diastolic BP (142.37/84.6 mmHg) compared to normotensive (123.71/76 mmHg) subjects, and indeed, the measurements of Bp were significantly different.

## 6. Discussion

Our study investigated the physiological, behavioral, and dietary characteristics associated with hypertension among Kenyan defence forces. The observed characteristics were consistent with those seen in patients with hypertension in developed western countries [20] and are in line with other studies on civilian population [21]. This is likely to raise a debate on the role of physical activity in maintaining health and specifically the cardiovascular health. Ordinarily, the military personnel ought to be physically fit, with healthy cardiovascular parameters because of their stringent healthy criteria, regular physical activity schedule, controlled military weight standards, and regular medical checkups and health awareness. Our findings, however, point to the fact that hypertension may be a resultant of interplay between multiple factors. 

We show that aging was associated with diagnosis of hypertensive disease consistent with other studies. For instance, a study from Cameroon found that the mean Bp and prevalence of hypertension increased with advanced age [8]. Other studies have also implicated the risk of developing hypertension to age, with significant increase occurring among 45–74 years age group [22]. Our participants were mainly males showing that military is male-dominated profession. 

Diagnosis of hypertension appeared to be lower among participants with tertiary compared to primary and secondary education. An inverse relationship between hypertension and level of education was depicted. This may be attributed to health awareness and practices among participants who attained higher education. Studies have shown that hypertension and its risk factors are relatively unknown to people of lower education level. Additionally, higher educational achievement has been associated with greater healthcare seeking behavior and awareness that may alleviate the risk factors associated with hypertension [23]. Well educated people are more knowledgeable, practice healthy behavior, and have superior nutritional education and practices and strong stress coping mechanism which may synergize in prevention of chronic diseases.

Alcohol consumption was related to diagnosis of hypertensive disease. Participants with hypertensive disease were more likely to consume beer or spirits. These findings are consistent with studies that have established a linear relationship between daily consumption of more than 3 drinks of alcohol and the increased risk of hypertension [24]. Recently, among the Spaniards, alcohol consumption specifically beer and spirits have been implicated with high incidence of hypertension [25]. The mechanisms of action of alcohol on cardiovascular system are well documented. Alcohol acts to augment the vasoconstriction caused by catecholamines and vasopressin which inhibits endothelium-dependent vasodilatation resulting in high blood pressure [26], whereas its reduction results in a dose-dependent decline in both systolic and diastolic Bp [27]. 

A direct relationship between current smoking and hypertension was demonstrated with rates among smokers compared to nonsmokers. The findings concur with those of Mancia and coworkers [28] who reported that smokers had higher ambulatory BP compared to nonsmokers, but cessation of smoking failed to directly reduce the BP. We established that participants who once smoked but stopped still developed hypertensive disease. The duration of smoking was directly related to diagnosis of hypertension despite smoking cessation. These findings are consistent with Wenzel and colleagues who established a similar pattern among Brazilian soldiers, in which prevalence of hypertension was higher among ex-smokers [29]. Several other studies have implicated current smoking to increased BP and the risk for hypertension with the underlying mechanism due the role of nicotine in activating sympathetic nervous system and the resultant arterial stiffness [30, 31].

Documented evidence strongly supports the effect of multiple dietary factors on BP. Our study showed a relationship between infrequent consumption and hypertension. The inverse relationship between fruits, vegetables consumption, and hypertension is well documented [25]. For instance, in the Dietary Approaches to Stop Hypertension (DASH) study, diet rich in fruits, vegetables, and low-fat dairy products with reduced saturated fat were shown to reduce systolic BP by 5 mmHg [32]. Furthermore, higher intake of fruits and vegetables was related to slow rise in systolic BP and diastolic BP over time regardless of age, weight, and other foods [33], and high-level consumption of fruits and vegetable has been implicated with lower risk of hypertension [34]. Vegetable and fruit dietary patterns are inversely associated with CVD mortality, whereas meat dietary pattern is directly associated with CVD or ischemic heart disease mortality [35]. 

The relationship between fruit and vegetable dietary pattern and the risk for hypertension may be explained biochemically. Fruits, vegetables, tubers, and legumes are flavonoid-rich food sources. Flavonoids are structurally diverse compounds that exhibit cardioprotective properties which include antioxidant, anti-inflammatory, and induction of apopttoic effects [36–39]. Studies have shown that flavonones and foods rich in flavonoids lower CVD mortality [40]. Oxidative stress which is a disturbance in the equilibrium status of prooxidant/antioxidant systems in intact cells has been proposed as the cause of hypertension [41]. The antioxidant activity and increased levels of reactive oxygen species scavenging conferred by flavonoid-rich foods such as fruits and vegetables may therefore be a plausible explanation on their hypertension reducing properties.

We have shown that the frequency of red meat consumption among hypertensive participants was slightly higher. There was a clear association between consuming red meat and hypertension. The observation is consistent with other findings which have established the role of diet low in fruits, high in fats, and red meat increases chances of hypertension and CVD [42]. Increased consumption of red meat and reduced intake of fruits among hypertensive participants in our study point toward the possible association and the risk for hypertensive disease. Among Cameroonian defense forces, it was also established that consumption of vegetables and fruits, tubers, and legumes was inversely associated with the prevalence of hypertension [43]. Similarly, a recent report showed an association between frequent meat and fat consumption with higher prevalence of hypertension in Sub-Saharan Africa [44].

The results showed an association between the type of work performed and the BP status. There was significant association between report on engagement in muscular endurance exercises and reduced chances of hypertension among participants. It is well established that physical inactivity is a risk factor for hypertension and other CVD, [45], and, therefore, exercise plays a critical role in maintaining optimum blood pressure. This is consistent with other studies, implicating sedentary lifestyle to risk of hypertension. Recently, physical activity and physical fitness were inversely related to incidence of hypertension [46]. Regular aerobic activity has been associated with reduced systolic blood pressure by an average of 6.9 mmHg and diastolic blood pressure of 4.9 mmHg [47]. 

Participants with hypertensive disease had higher anthropometric (BMI and WHR) parameters, consistent with the strong association between higher BMI and increased risk of hypertension [48]. The association has consistently been associated with central obesity (an indicator of higher waist-to-hip ratio) and abnormal lipid profiles which are key risk factors in development of hypertension [49].

Finally, we demonstrated the association between hypertension and participation in peace missions. This may be attributed to stress resulting from change of climatic conditions, combat exposure, and separation from family and friends. High incidence of hypertension has been reported among soldiers deployed to combat zones [50]. Combat exposure elicits psychological stress due to adjustment to various sociocultural background, engagement with enemy forces, encounter with wounded civilians or soldiers and maimed bodies, and also loss of colleagues, which is a fact that may explain the high prevalence of hypertension among the armed forces [50]. 

Our study holds several strengths. This is the most recent study to investigate the physiological, behavioral, and dietary characteristics associated with hypertension among KDF. It, therefore, has a great public health importance with potential relevance for other armed forces and disciplined forces in many developing countries. The study was well structured to comprehensively capture sociodemographic, lifestyle characteristics, anthropometric measures, and dietary patterns as well as physical activity levels. We combined both survey questionnaire and objective measurement of physiological and anthropometric parameters designed to take into account complex interactions among different components and their cumulative influences on hypertension among the military personnel. More research is required to establish whether prediction rules and/or risk scores may be used to identify at-risk individuals for preventive strategy as well as targeted specific behavioral interventions among this population. 

This study holds a number of limitations. The study population may not be representative of the entire armed forces in Kenya because of the small sample. The participants were mainly recruited from military institutions around Nairobi through AFMH where they had sought treatment. They had participated voluntarily, and this may have captured a population with a greater concern and awareness for their health therefore affecting generalizability. Furthermore, the assessment of smoking, alcohol consumption, dietary, and activity levels were retrospective; therefore, recall bias may not have been completely eliminated. However, such a bias may not have been substantial as this method served to collect information from the entire study population in the same manner, and the data were obtained before the screening. The study failed to compare different variables on the basis of ranks which may have missed critical analysis on which ranks of the military are highly affected by the hypertension, an important guide to intervention. The study also missed to capture salt intake and lipids profiles among Kenya military which are critical components in risk assessment. Our study design failed to establish whether the combination of smoking, less physical activity, and stressful work condition results in more severe disease. Finally, this was a cross-sectional study and because of the design, the causal relationship between characteristics and diagnosis hypertension may not be strongly established.

In conclusion, we underscore the role of lifestyle, social habits such as smoking, alcohol consumption, and physical inactivity, higher anthropometric parameters (BMI and WHR), and diet rich in red meat and low in fruit for their association with hypertension. The findings of the physiological, behavioral, and dietary characteristics among Kenya defence forces mimic the main risk factors and characteristics for CVD among patients in the western developed countries whose lifestyle adoption is happening fast in low and middle-income countries. 

## Figures and Tables

**Table 1:** Selected demographic, social behavior, and dietary characteristics of the study population by hypertension status.

Variable	Hypertensive *N* (%)	Normotensive *N* (%)	OR (95% CI)	P value
Age (years)				
18 to 28	11 (6.47)	12 (7.06)	1.00	
29 to 38	19 (11.12)	51 (30.00)	0.40 (0.15–1.07)	
39 to 48	75 (44.12)	84 (49.41)	0.97 (0.40–2.33)	
49 to 58	65 (38.24)	23 (13.53)	3.96 (1.2–7.94)	<0.05
Gender				
Male	161 (94.7)	151 (88.8)	1.00	
Female	9 (5.3)	19 (11.2)	0.44 (0.19–1.01)	<0.05
Education level				
Primary	29 (17.0)	3 (1.76)	1.00	<0.05
Secondary	119 (70)	65 (38.24)	0.19 (0.06–0.50)	
Tertiary	22 (11.18)	102 (58.24)	0.10 (0.01–0.75)	
University	3 (1.76)	3 (1.76)	0.10 (0.01–0.75)	
Alcohol consumption				
Yes	107 (62.94)	88 (52.07)	1.56 (0.41–1.98)	0.043
No	63 (37.06)	81 (47.93)	0.64 (0.1–0.98)	
Frequency				
Daily	14 (12.50)	2 (2.27)	6.29 (0.6–6.77)	<0.01
1–3 days/week	70 (62.50)	29 (32.95)	3.39 (0.2–3.43)	<0.05
1–3 days/month	22 (19.64)	39 (44.32)	0.31 (0.05–0.96)	
Once/month	6 (5.36)	18 (20.45)	0.22 (0.04–0.67)	
Type of alcohol				
Beer	93 (83.04)	66 (74.16)	1.54 (0.3–1.6)	<0.05
Wine	6 (5.36)	13 (14.61)	0.32 (0.2–0.52)	
Spirit	15 (11.61)	10 (11.24)	1.2 (0.04–1.3)	<0.05
Smoking				
Yes	18 (10.59)	7 (4.12)	1.00	0.027
No	152 (89.41)	163 (95.88)	0.17 (0.14–0.89)	
Dietary patterns				
Frequency of eating fruits				
Daily	37 (21.76)	45 (26.47)	1.00	
5-6 days/week	10 (5.88)	43 (25.29)	0.08 (0.02–0.63)	0.002
1–4 days/week	123 (72.35)	82 (48.24)	1.82 (1.08–3.05)	0.023
Frequency of eating vegetables				
Daily	108 (64.12)	112 (65.88)	1.00	
5-6 days/week	19 (11.18)	33 (19.41)	0.59 (0.31–1.10)	0.099
1–4 days/week	42 (24.71)	25 (14.71)	1.72 (0.98–3.02)	0.056
Frequency of eating red meat				
Daily	113 (66.47)	78 (45.88)	1.00	
3 times/week	44 (25.88)	63 (37.06)	0.48 (0.29–0.78)	0.003
Weekly	11 (6.47)	27 (15.88)	0.28 (0.13–0.60)	0.001
Monthly	2 (1.18)	2 (1.18)	0.69 (0.09–5.0)	0.714

Values adjusted for age, gender, and educational level.

**Table 2:** Selected peace mission participation, activity level, anthropometrics, and physiological characteristics of the study population by hypertension status.

Participation in peace mission	Hypertensive, *N* (%)	Normotensive *N* (%)	*χ* ^2^	*P* value
Yes	99 (68.28)	46 (31.72)	145 (100)	
No	70 (36.08)	124 (63.92)	194 (100)	
	*χ* ^2^ = 34.40; df = 1; *P* < 0.001			
Muscular endurance exercise				
Yes	63 (37.06)	138 (81.18)	1.00	
No	107 (62.94)	32 (18.82)	2.53	<0.01
	*χ* ^2^ = 9.34; df = 2; *P* = 0.009			
Anthropometric measurements				
BMI				
Normal	35 (20.71)	116 (68.24)	28.86	<0.01
Overweight	101 (59.76)	48 (28.24)		
Obese	33 (19.53)	6 (3.53)		
Waist hip ratio				
Normal	114 (67.06)	166 (97.65)	15.68	0.01
Central obesity	56 (32.94)	4 (2.35)		
Heart rate	79.91	73.89	6.02	0.05
Blood pressure				
Mean systolic Bp (mmHg)	142.37	123.71	18.66	<0.05
Mean diastolic Bp (mmHg)	84.60	75.99	8.61	<0.05

Abbreviations: BMI: body mass index; BP: blood pressure; mmHg: millimeters of mercury.
